# Techniques for Repair of the Thumb Ulnar Collateral Ligament Without Suture Anchors

**DOI:** 10.7759/cureus.76892

**Published:** 2025-01-04

**Authors:** Varun Arvind, Robert J Strauch

**Affiliations:** 1 Orthopedics, Columbia University College of Physicians and Surgeons, New York, USA; 2 Orthopedic Surgery, Columbia University College of Physicians and Surgeons, New York, USA

**Keywords:** anchor, anchor free fixation, stener lesion, thumb, ucl, ucl injury

## Abstract

Thumb ulnar collateral ligament injuries are common and can require surgical repair to restore thumb stability. Techniques that use suture anchors are challenging in patients with poor bone stock or cortical defect at the attachment site. Therefore, we describe anchor-less techniques for ulnar collateral ligament repair, with one new technique simplifying repair. We then report the clinical results for one patient treated with this new technique.

## Introduction

Gamekeeper's thumb, also known as skier's thumb, is a common injury that affects the ulnar collateral ligament (UCL) of the thumb metacarpophalangeal (MCP) joint with an estimated incidence of approximately 50 patients per 100,000 emergency department visits [1]. This injury typically results from a forceful abduction or hyperextension of the thumb, leading to a partial or complete tear of the UCL [1]. For partial UCL tears, non-surgical treatment may be sufficient with cast immobilization for four to six weeks followed by range-of-motion exercises [2]. Operative treatment of UCL tears includes primary ligament repair or reconstruction. Typically, the UCL ruptures from the base of the proximal phalanx, and primary repair may be performed with suture and drill holes vs suture anchors [1,3]. As per Jupiter et al., primary repair requires a two-hole drilling technique that is technically difficult and not without complications [4]. More recently, suture anchor-aided fixation has gained popularity for relative technical ease and equivalent outcomes [5-8]. However, management of UCL injury involving an avulsion fracture of the base of the proximal phalanx often requires excision of the small bony fragment. Repairing the UCL to an open bony bed poses challenges with respect to using a suture anchor as many suture anchors require intact cortical bone to achieve adequate purchase. A cortical defect, with exposed cancellous bone at the anatomic site of ligament reattachment, therefore obviates the use of many types of suture anchors. Moreover, in patients with poor bone quality or inadequate bone stock sutures, anchor fixation may pose additional challenges.

Therefore, surgical techniques for the treatment of UCL injuries that circumvent the use of suture anchors present an attractive alternative treatment option for the treatment of gamekeeper’s thumb when there is a bony cavity making suture anchor use difficult. Such techniques allow anatomic reattachment of the UCL to its origin on the base of the proximal phalanx and decrease the cost of repair by forgoing suture anchor use. In this paper, we present the case of a patient with a UCL tear with a Stener lesion of the left thumb, repaired using a suture-anchorless method. We further review surgical techniques that allow effective re-attachment and healing of the UCL without the use of suture anchors and introduce one new simple technique that provides anatomic repair of the UCL with a one-hole drilling technique.

## Case presentation

**Figure 1:**
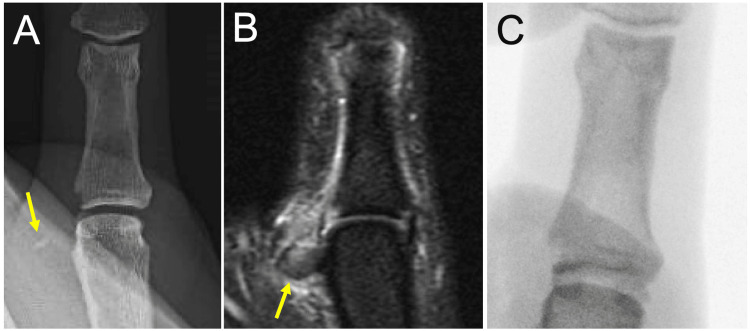
X-rays (A) demonstrating bony UCL avulsion (yellow arrow), with Stener lesion on MRI (B, yellow arrow). Intraoperative fluoroscopy image following repair (C). UCL, ulnar collateral ligament; MRI, magnetic resonance imaging.

**Figure 2:**
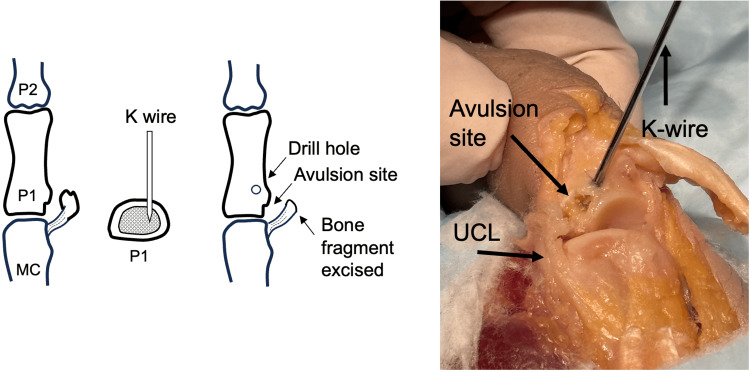
Unicortical tunnel drill hole placement. UCL, ulnar collateral ligament; MC, metacarpal. Image credit: VA.

**Figure 3:**
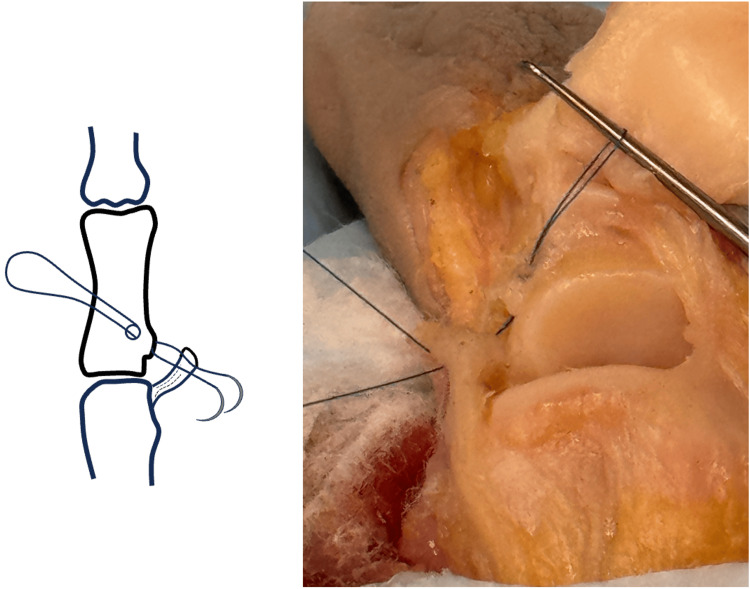
Suture is passed through the UCL, with suture loop formed. UCL, ulnar collateral ligament. Image credit: VA.

**Figure 4:**
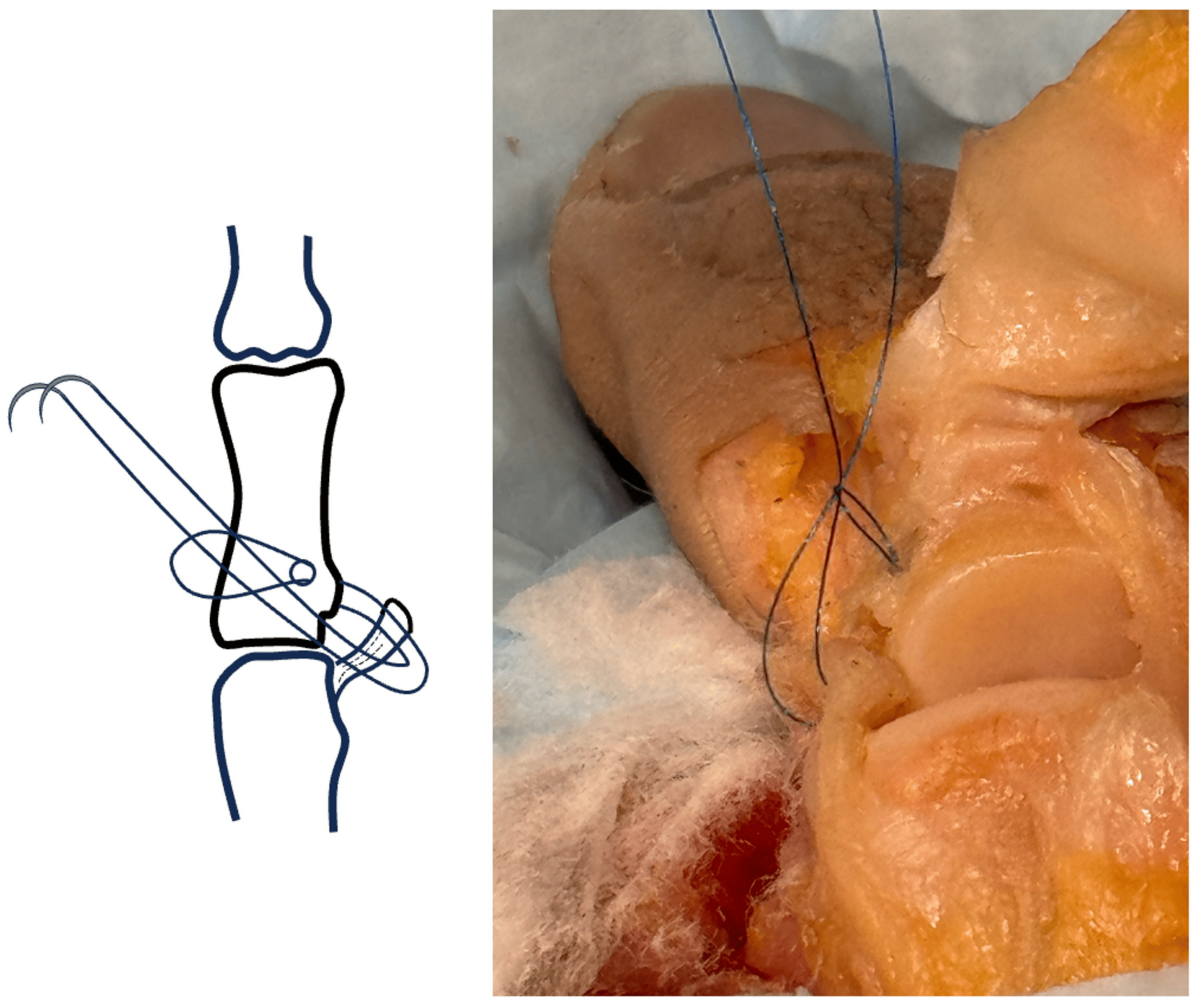
Lark knot created by passing suture needles through a suture loop over the bone bridge. Image credit: VA.

**Figure 5:**
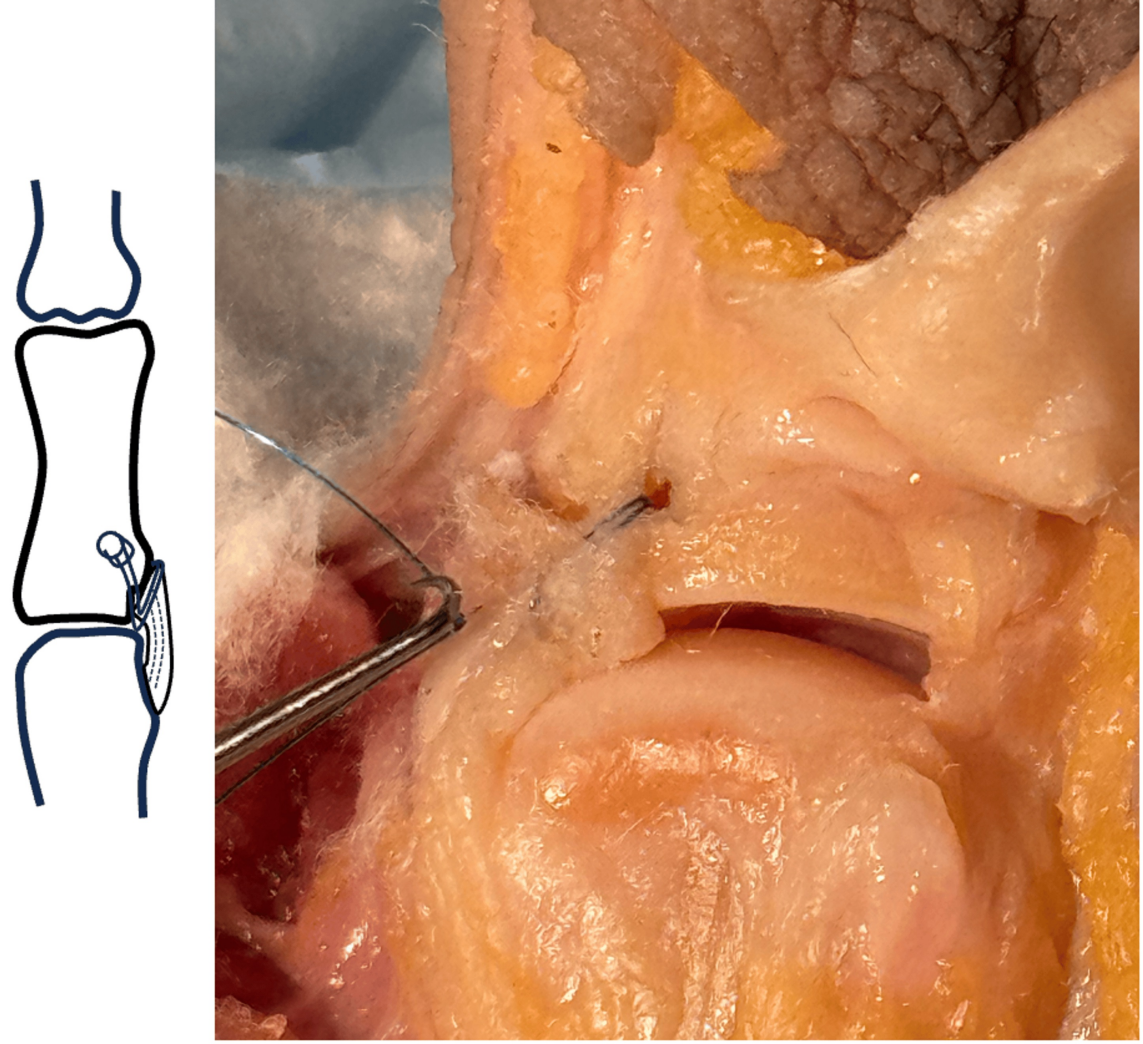
UCL is gently advanced into the avulsion site and the suture is fixed to UCL to secure repair. UCL, ulnar collateral ligament. Image credit: VA.

A 49-year-old woman with no significant medical history presented to the hospital one week following a fall. The mechanism of injury was left thumb abduction while bracing her fall and sustained with a gamekeeper’s injury of the left thumb. On examination, initially, the patient presented with swelling and bruising over the left thumb with x-rays demonstrating a displaced fracture at the insertion of the UCL to the base of the proximal phalanx of the left thumb. Subsequent magnetic resonance imaging (MRI) revealed an avulsed complete UCL tear with a Stener lesion with a small bony fragment attached to the UCL (Figure 1A, 1B). On physical examination, the range of motion of the left thumb was limited to 20 degrees compared to 45 degrees on the right. The left thumb was tender with bruising and a bump on the ulnar side of the thumb MCP joint consistent with the Stener lesion. Intraoperatively, we incised the abductor aponeurosis, and the Stener lesion was confirmed. The small bone fragment was excised from the UCL, and a repair was performed per the one-hole drill technique described below (Figures 2-5). Briefly, an incision was made on the ulnar side of the left thumb MCP joint. The superficial radial nerve was identified and protected. The Stener lesion was visible proximal to the adductor tendon. The fracture bed was debrided and the tiny avulsion fragment was excised from the UCL. A 0.045 K-wire was used to drill a hole dorsal to the fractured bed and a double-armed 5-0 Prolene suture was passed through the drill hole creating a suture loop and then through the UCL. The suture ends were then passed through the sutured loop, and the UCL was cinched to the fractured bed and secured (Figures 2-5). The skin was closed and the hand was placed in a thumb spica splint with four weeks of postoperative immobilization. At the three-month follow-up, the patient was recovering appropriately with an improved range of motion of the left thumb MCP joint to 45 degrees.

Anatomy

The thumb UCL plays a crucial role in the stability and function of the thumb, particularly at the MCP joint. The UCL is vital for the precise movements of the thumb including grasping, pinching, and manipulating objects. The primary function of the UCL is to provide stability to the thumb's MCP joint against valgus stress. UCL insufficiency can present with key-pinch and tip-pinch weakness [9]. The thumb UCL is composed of two main parts, the proper and accessory collateral ligaments. The proper collateral ligament is the main component of the UCL, originating from the metacarpal head and inserted into the base of the proximal phalanx of the thumb. It is taut during flexion of the MCP joint, providing stability throughout the joint's range of motion [2]. The accessory collateral ligament of the UCL runs palmar to the proper ligament. It originates and inserts in a similar manner but is primarily taut when the MCP joint is in extension [2]. The accessory ligament aids in stabilizing the joint, particularly when the thumb is extended.

During primary UCL repair, reattachment of the proper UCL must be performed anatomically to the isometric point [7]. Several studies have reported decreased motion and persistent laxity following non-anatomic UCL reattachment [10,11]. Palmar and dorsal reattachment of the UCL increased radial deviation [2]. Therefore, the use of bone tunnels or suture anchors that result in non-anatomic reattachment may result in defects in thumb function.

Indications/contraindications

Operative treatment for an acute gamekeeper's thumb, also known as a thumb UCL injury, is indicated for acute Grade 3 injuries, which present as joint instability without endpoint and a 30-35° of joint space opening or 10-15° greater than a non-injured contralateral thumb [1]. It is also indicated with concomitant Stener lesions, where the avulsed ligament is displaced with interposition of the adductor pollicis aponeurosis obstructing UCL reattachment [1,12]. In situations where there is a small avulsion fragment at the ulnar base of the proximal phalanx, an MRI or ultrasound can determine if the UCL is attached to the fragment (occasionally the UCL will itself be avulsed from the small fragment of bone). If the bony fragment is large and malreduced, repair of the fragment can be undertaken. Usually, the fragment is small and minimally displaced, in which case the injury can be treated nonoperatively with immobilization for a month followed by the range of motion assuming the UCL is attached to the fragment. In the more unusual case where the fragment is quite displaced, or where the UCL has avulsed from the fragment, surgical repair of the UCL can be performed with excision of the small fragment.

Relative contraindications to repair include patients with partial tears which heal with immobilization. Contraindications include degenerative joint disease of the MCP joint which may benefit from arthrodesis. Additionally, the integrity of the remaining stabilizers of the thumb MCP must be evaluated. Insufficiency of these structures with the inability to reconstruct may necessitate arthrodesis [1].

Technique

An incision is made ulnarly over the thumb MCP joint. Dissection is taken through the subcutaneous tissue and careful identification and protection of the superficial radial nerve is performed.

Deeper dissection is continued down to the adductor aponeurosis which is incised, leaving enough tissue on either end for repair. The joint capsule is incised. With reflection of the aponeurosis, the MCP joint is visualized and the UCL stump is identified; invariably, the ligament ruptures distally leaving its proximal attachment intact. In the case of a small avulsion fracture, the bony fragment is shelled out from the distal end of the UCL with a scalpel.

A unicortical tunnel is prepared dorsal and distally to the UCL avulsion site. This is performed using a 0.045 inch K-wire (Figure 2). The tunnel should be in continuity with the avulsion site with a small bony bridge between the two, such that a suture can be passed through the tunnel for later UCL reattachment.

Using a double-armed 5-0 Prolene or a similar suture, both needles are passed through the tunnel into the avulsion site creating a loop that protrudes from the K-wire hole (Figure 3). The loop can be secured using a skin hook to prevent accidental suture pullout through the tunnel. The suture needles are then passed through the same side of the distal end of the UCL stump in a non-locking fashion to allow free gliding of the suture through the ligament.

The needles are then passed through the suture loop to form a Lark’s knot (Figure 4). Once through the loop, the Lark’s knot is gently cinched to dock the UCL into the avulsion site.

Once the ligament is approximated, the suture needles are then passed through the UCL with a locking knot to fix the UCL to the avulsion site (Figure 5). Under direct visualization, the repair may be gently tested. The wound is irrigated and the capsule reapproximated over the graft and the skin is closed according to the surgeon's preference.

**Figure 6:**
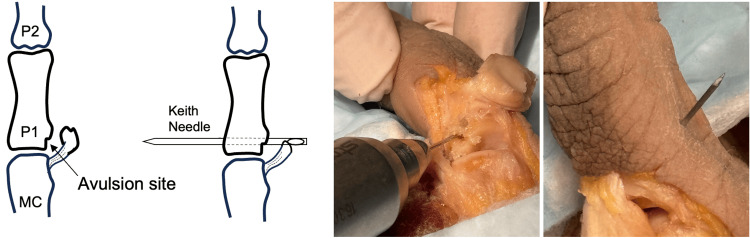
A transosseous tunnel through the avulsion site and the distal cortex is created using a Keith needle. MC, metacarpal. Image credit: VA.

An additional, more traditional UCL repair technique may be performed. Following dissection and debridement of the UCL, a Keith needle is loaded into a pin driver and driven through the avulsion site through the distal cortex (Figure 6).

**Figure 7:**
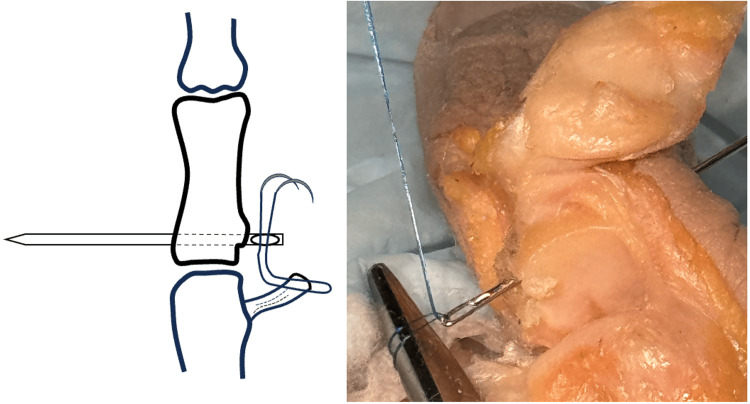
Suture is placed into the UCL and the suture needles are passed through the needle eyelet. UCL, ulnar collateral ligament. Image credit: VA.

Using a double-armed 5-0 Prolene suture or similar, a suture is placed into the end of the UCL. The needles are then passed through the Keith needle eyelet (Figure 7). The Keith needle is then inserted into the K-wire driver and driven through the bony bed to the radial cortex of the proximal phalanx and out of the skin.

**Figure 8:**
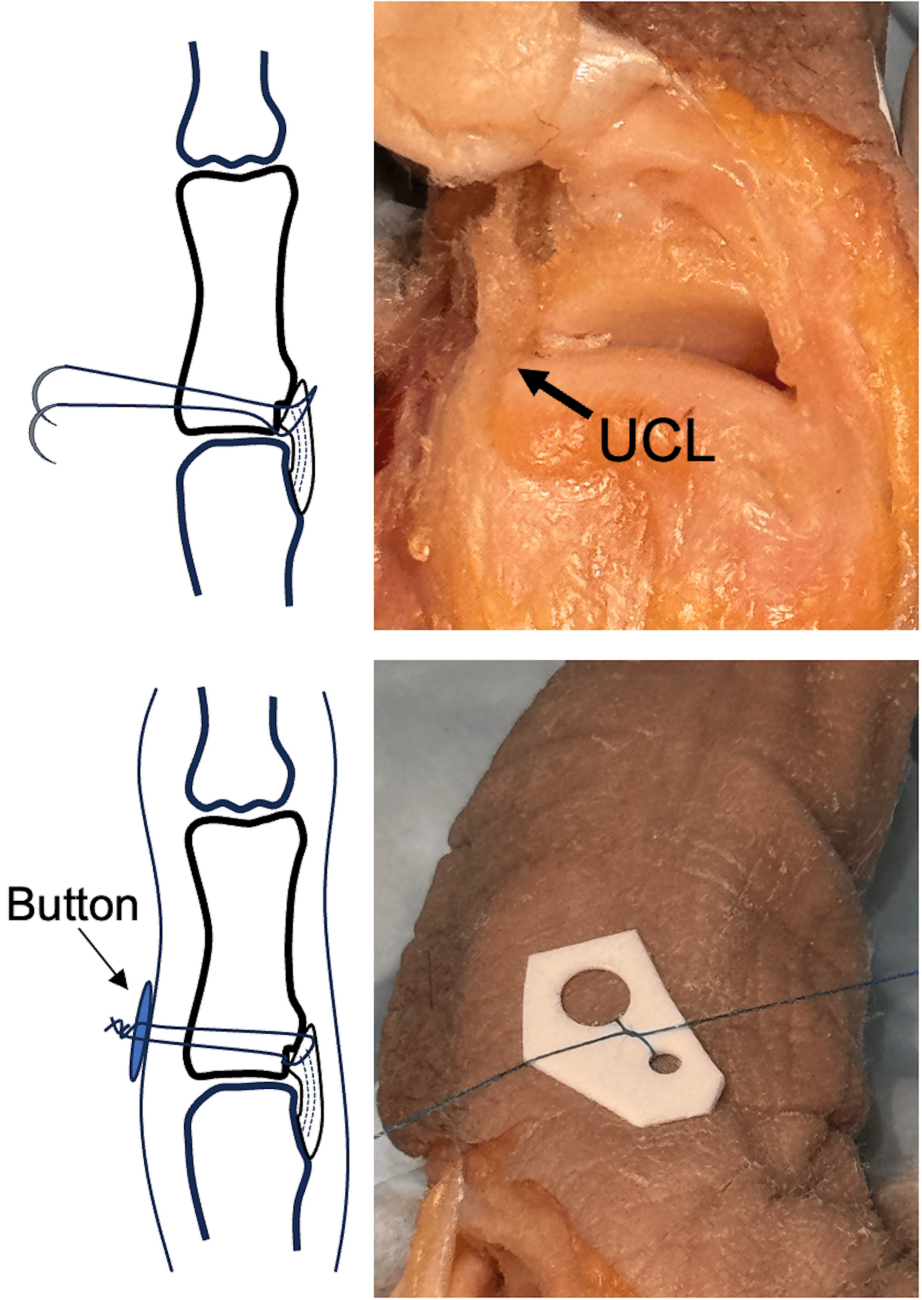
Suture is passed through the tunnel by advancing the needle and secured using an external button. UCL, ulnar collateral ligament. Image credit: VA.

The suture is then knotted down over a button or a pledget on the skin to secure the repair (Figure 8). As before, the repair is gently tested under direct visualization and the wound is irrigated followed by closure of the capsule and skin. Alternatively, a small skin incision can be made, and the suture ends sutured into the proximal phalanx periosteum to avoid the external button.

**Figure 9:**
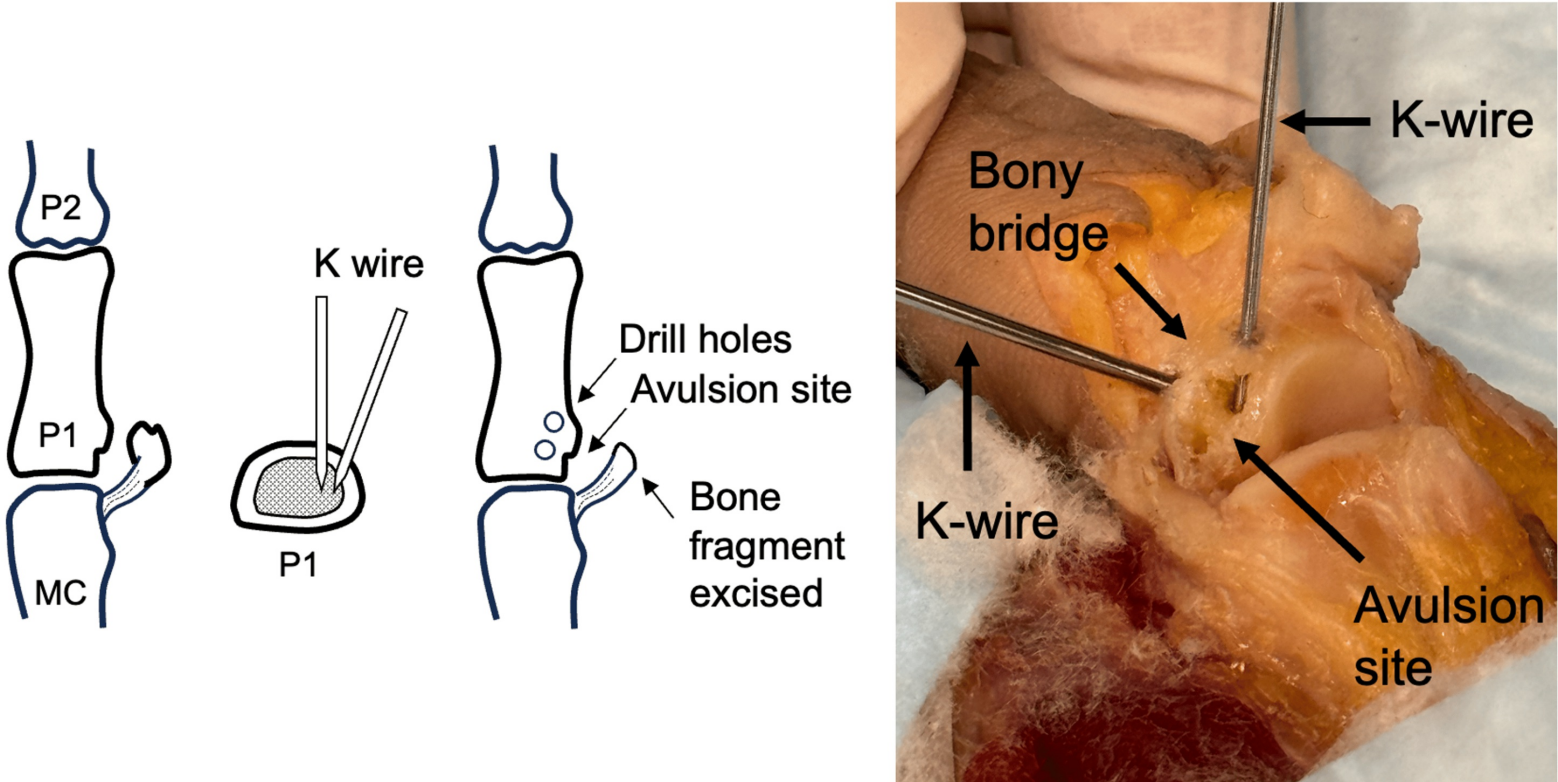
Two-hole drilling technique. MC, metacarpal. Image credit: VA.

Finally, we present the more standard two-hole drilling technique. Two unicortical tunnels are prepared dorsal and distally to the UCL avulsion site using a 0.045 inch K-wire (Figure 9). Careful drilling of tunnels should be performed to ensure continuity of tunnels, with a small bony bridge between either tunnel.

**Figure 10:**
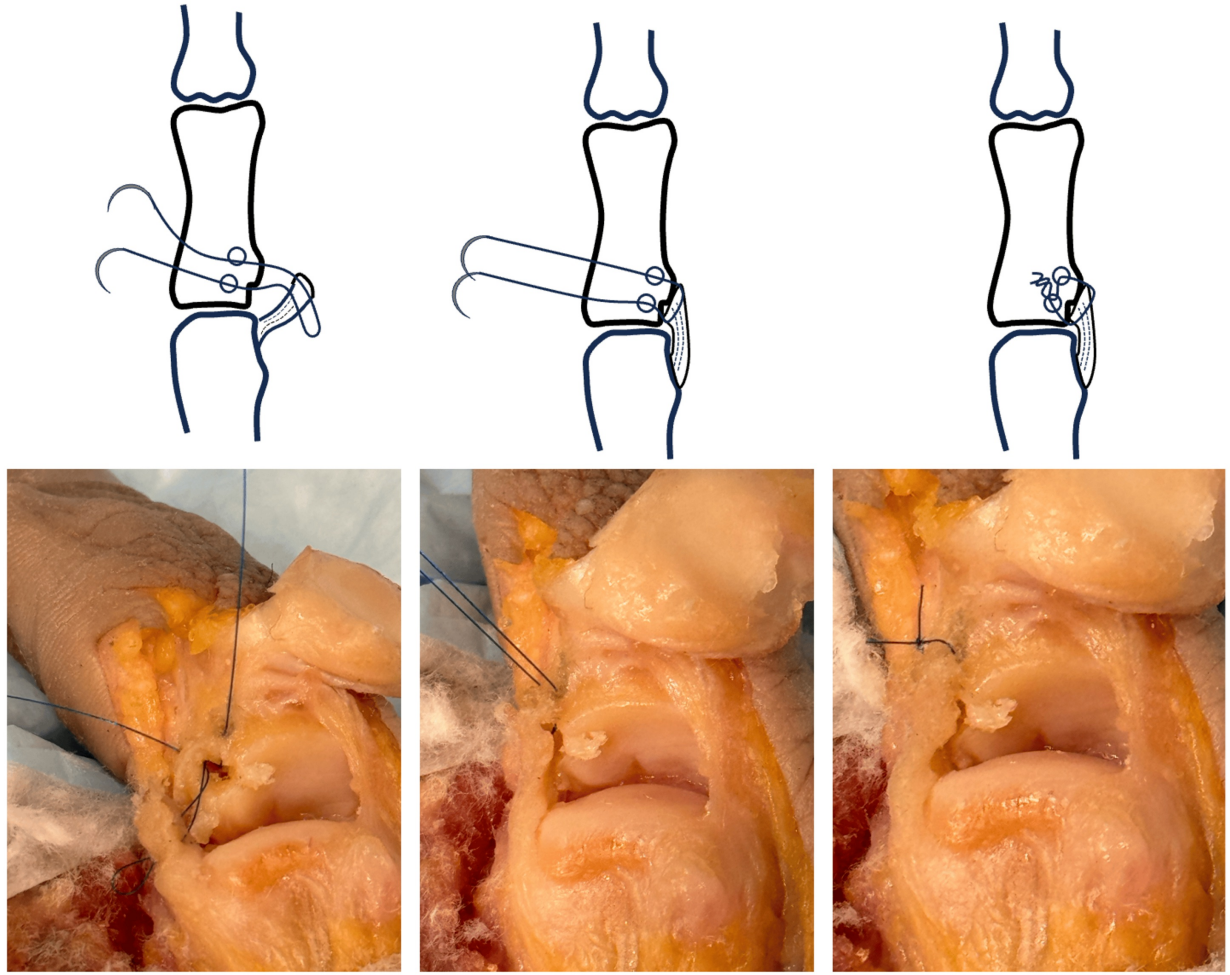
The suture is passed through both drill holes and the tendon. The suture is then knotted down over the bony bridge, advancing the UCL tendon to the avulsion site. UCL, ulnar collateral ligament. Image credit: VA.

Using a double-armed 5-0 Prolene, or similar suture, the needle is threaded through one drill hole, then through the UCL stump. The suture needle is then passed back through the second drill hole (Figure 10). The suture is then knotted down over the bony bridge thereby advancing and securing the UCL to the avulsion site.

## Discussion

Thumb UCL repair is important for restoring stability and function in cases of severe injuries, such as a gamekeeper's or a skier's thumb. Anatomical reattachment of the UCL is essential to maintain the thumb's range of motion and resist valgus stress, ensuring effective pinch and grip functions.

Surgical repair of UCL avulsion injuries results in excellent functional outcomes. Pichora et al. found that greater than 90% of patients achieved good or satisfactory results following surgical repair of UCL injuries [12]. Following repair, pinch strength recovered to 89% of the contralateral thumb at one year postoperatively [12]. Among patients with thumb UCL avulsion fractures, 89.2% of patients had similarly good outcomes following surgical repair [13]. In contrast, among patients with milder displacement (<2 mm) who were treated nonoperatively, 11% required conversion to surgical repair [13]. In a cross-sectional study by Mikhail et al., 97% of surgeons utilized a bone anchor to reconstruct the UCL attachment for repair [14].

Suture anchors offer technical simplicity but face limitations in patients with poor bone quality or cortical defects and also lead to increased costs. Anchor-less techniques are much less common with limited long-term studies that report the long-term outcomes in patients with anchor-less UCL repairs. The anchor-less repair technique presented here presents a viable alternative to suture-based UCL repair techniques, reducing hardware-related costs and complications. Here we present several simplified anchor-less methods for thumb UCL repair that hold promise in improving surgical efficiency and accessibility. Future comparative studies are needed to establish the long-term efficacy and broader applicability of these techniques.

After UCL repair, the thumb is immobilized in a thumb spica splint for six weeks. During this time interphalangeal joint motion is encouraged. At four weeks, the splint is removed, and then guided hand therapy is encouraged to regain motion. A removable splint can be used to protect the repair. Gripping and pinching can be resumed at 10-12 weeks, with forceful gripping resumed at 12 weeks.

The primary advantage of non-suture anchor fixation in the setting of excision of a bony fragment is that many anchors require intact cortical bone and cannot be used in this situation. Further advantages include cost savings and avoidance of complications associated with suture anchor fixation. Functional outcomes are expected to be similar to suture anchor reconstruction techniques.

Complications of this technique are similar to other operative treatments for UCL tears. Anatomic structures at risk during exposure include the superficial branch of the radial nerve. Chuter reported a complication rate of 17% following UCL repair, with the most common causes being neurapraxia (6.3%) and subjective stiffness (3.9%) [15]. 

Careful dissection, delicate handling of soft tissues, and compliance with postoperative hand therapy are important mitigating factors to decrease complications. Recurrent instability, thumb laxity, or stiffness may arise due to non-anatomic reattachment or improper tensioning of the ligament. Pullout sutures using the transosseous fixation likely present an increased risk of infection and skin dimpling due to button tensioning.

## Conclusions

UCL repair without suture anchors represents a viable approach to address the challenges faced in cases of poor bone stock or cortical defects. By offering alternative methods, including a novel one-hole drilling technique, this study highlights the importance of anatomical reattachment to maintain thumb stability and function. The described technique simplifies the surgical procedure while reducing cost and avoiding complications associated with suture anchor use. The clinical case presented here demonstrates its efficacy, suggesting comparable outcomes to conventional techniques while addressing limitations such as inadequate cortical bone.
